# [Corrigendum] p57^KIP2^-mediated inhibition of human trophoblast apoptosis and promotion of invasion *in vitro*

**DOI:** 10.3892/ijmm.2026.5754

**Published:** 2026-02-04

**Authors:** Guo-Qian He, Guang-Yu Liu, Wen-Ming Xu, Hui-Juan Liao, Xing-Hui Liu, Guo-Lin He

Int J Mol Med 44: 281-290, 2019; DOI: 10.3892/ijmm.2019.4175

Following the publication of the above article, an interested reader drew to the authors' attention that, concerning the Transwell migration assay images shown in Fig. 6 on p. 287, the data panels for figure parts 6E (the DMSO experiment) and 6G (the pcDNA3.1+DMSO experiment) contained strikingly similar data, albeit with different sizing of the images, suggesting that these data had been derived from the same original source.

Upon investigating this figure, the authors realized that this figure had inadvertently been assembled incorrectly: The data panel for the DMSO group in the HTR-8/SVneo cell migration assay (Fig. 6E) had been duplicated from the correctly displayed pcDNA3.1+DMSO group panel. The revised version of Fig. 6, now showing the correct data panel for Fig. 6E, is shown on the next page. The authors confirm that the error associated with this figure did not have any significant impact on either the results or the conclusions reported in this study, and all the authors agree with the publication of this Corrigendum. The authors are grateful to the Editor of *International Journal of Molecular Medicine* for allowing them the opportunity to publish this Corrigendum; furthermore, they apologize to the readership of the Journal for any inconvenience caused.

## Figures and Tables

**Figure 6:**
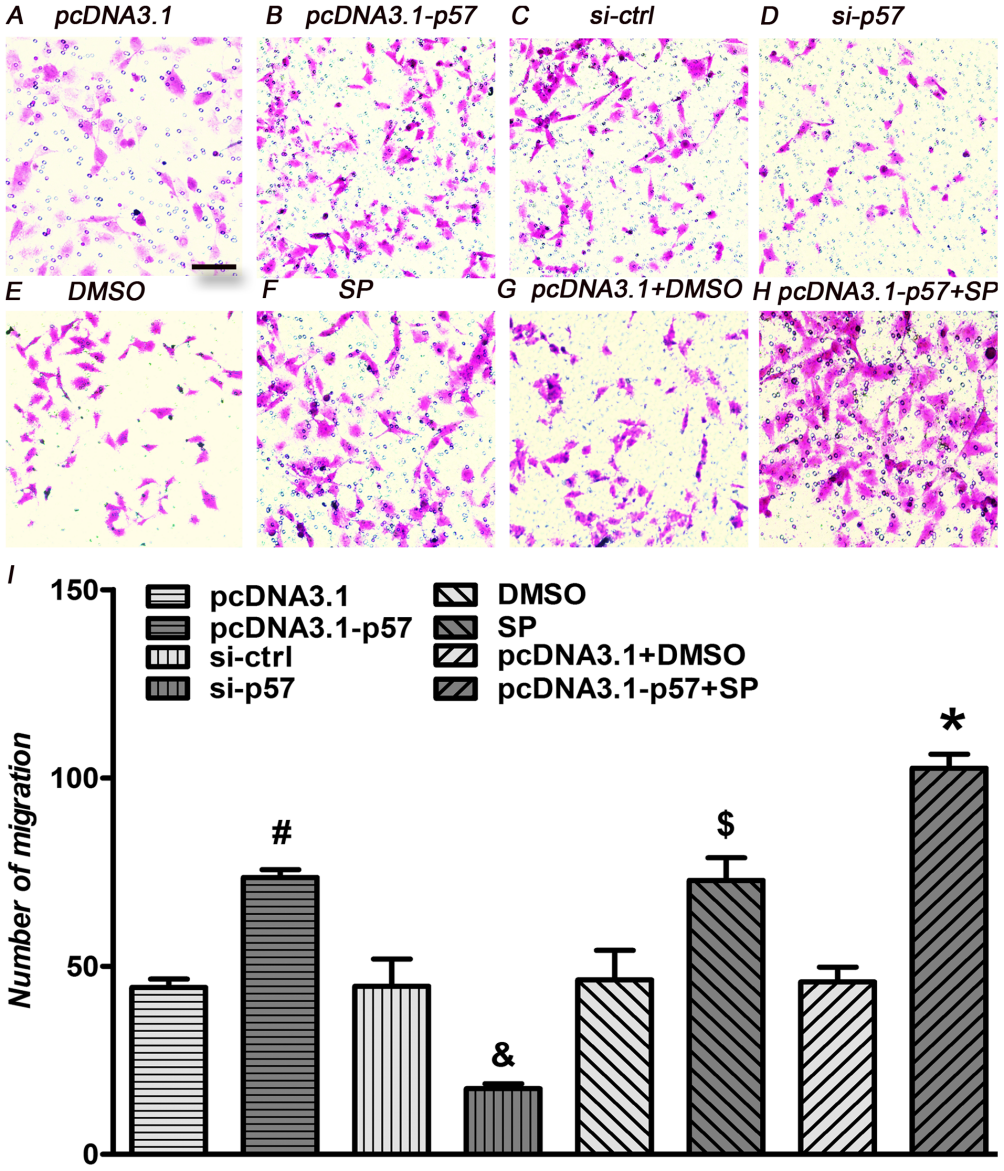
p57KIP2 and JNK inhibitor affect cell migration under hypoxic conditions. HTR-8/SVneo cells were transfected with pcDNA3.1-p57 or pcDNA3.1, treated with JNK inhibitor or DMSO, or a combination of both and p57KIP2 knockdown was achieved by transfection with si-p57; all procedures were followed by hypoxia for 24 h. Microscopy images if the Transwell migration assay for (A) pcDNA3.1, (B) pcDNA3.1-p57, (C) si-ctrl and (D) si-p57 transfected cells, for (E) DMSO and (F) SP treated cells, and for (G) pcDNA3.1 transfected and DMSO and (H) pcDNA3.1-p57 transfected and SP treated cells; magnification, ×20; scale bar, 10 *μ*m. (I) Number of migrated cells. ^#^P<0.05 vs. pcDNA3.1; ^&^P<0.05 vs. si-ctrl; ^$^P<0.05 vs. DMSO; ^*^P<0.05 vs. pcDNA3.1+DMSO. si-ctrl, siRNA control; si-p58, siRNA targeting p57KIP2; KIP, kinase inhibitory protein 2; SP, JNK inhibitor SP600125.

